# Neuroendocrine Breast Cancer-Associated Ectopic Adrenocorticotropic Hormone Syndrome Requiring Bilateral Adrenalectomy †

**DOI:** 10.3390/curroncol32040205

**Published:** 2025-03-31

**Authors:** Kala Hickey, Hannah Yaremko, Christine Orr, David Pace

**Affiliations:** 1 Department of General Surgery, Faculty of Medicine, Memorial University, St. John’s, NL A1B 3V6, Canada; 2 Faculty of Medicine, Memorial University, St. John’s, NL A1B 3V6, Canada; 3 Department of Medicine, Faculty of Medicine, Memorial University, St. John’s, NL A1B 3V6, Canada

**Keywords:** breast, neuroendocrine, ectopic adrenocorticotropic hormone syndrome, adrenalectomy, Cushing’s syndrome

## Abstract

Ectopic adrenocorticotropic hormone syndrome (EAS) occurs when a tumor develops neuroendocrine differentiation with the secretion of ACTH resulting in hypercortisolism and possibly Cushing’s syndrome (CS). Only 5–10% of CS cases are attributed to EAS; of these, breast tumors comprise less than 1%. Two known variants of breast neuroendocrine tumors include neuroendocrine-differentiated carcinoma and ductal carcinoma with neuroendocrine features. Currently, guidelines for treatment are limited and EAS is associated with significant morbidity and mortality. A 39-year-old female presented with a rapidly enlarging breast mass. Biopsy demonstrated invasive poorly differentiated breast carcinoma with high-grade neuroendocrine features and necrosis. Staging at diagnosis confirmed metastatic disease of the liver and bone. First-line chemotherapy (Cisplatin/Etoposide/Durvalumab) was initiated with evidence of disease progression after four cycles. Given a poor response to therapy, a simple mastectomy was performed for local control and complete pathologic analysis, demonstrating high-grade neuroendocrine carcinoma with large-cell features. Second-line therapy (Adriamycin/Cyclophosphamide) was initiated for three cycles after which the patient required admission for severe and refractory hypokalemia. Workup confirmed elevated ACTH consistent with paraneoplastic EAS and further evidence of disease progression. Third-line therapy (Nab-Paclitaxel) was initiated, and genetic testing was completed, confirming the PIK3 mutation, for which access to Alpelisib therapy was requested. Given symptoms of progressive severe CS with significant liver disease limiting medical therapies, the patient underwent urgent bilateral laparoscopic adrenalectomy after which she was able to be discharged home while awaiting additional systemic therapy. EAS resulting in CS secondary to breast neuroendocrine carcinoma is a rare and challenging diagnosis. Further research is needed to inform treatment guidelines to improve outcomes. While patient survival is dependent upon the underlying disease process, laparoscopic bilateral adrenalectomy is an accepted, definitive treatment option.

## 1. Introduction

Cushing’s syndrome (CS) refers to the multiple morbidities that result from abnormally elevated glucocorticoids, either through exposure to exogenous corticosteroids or endogenous cortisol [1]. Patients with CS experience multiple resultant cardiovascular and metabolic complications, including hypertension, hyperlipidemia, insulin resistance, obesity, thrombosis, and impaired immune function [2,3]. These comorbidities are associated with increased mortality, often as a result of cardiovascular complications [4].

Endogenous CS is subclassified into adrenocorticotropic hormone (ACTH)-dependent and ACTH-independent causes, which make up 80–85% and 15–20% of cases, respectively [2]. The vast majority of ACTH-dependent cases are due to Cushing’s disease, which is caused by the over-secretion of ACTH from a pituitary adenoma [5]. However, 10–20% of ACTH-dependent cases and 5–10% of all CS cases are attributable to ectopic ACTH syndrome (EAS) [6,7].

EAS is a paraneoplastic syndrome wherein a non-pituitary tumor secretes significant levels of ACTH such that a state of endogenous hypercortisolism arises [8]. The mechanism behind this is thought to be related to the abnormal hypomethylation of the pro-opiomelanocortin (POMC) promoter and thus the overexpression of POMC gene products resulting in increased ACTH secretion [9]. Classically, ectopic ACTH-producing tumors described in the 1960s were highly malignant; however, more recently, slow-growing neuroendocrine tumors (NETs) have been reported with increasing frequency (up to 90%) [10].

NETs are a heterogeneous group of neoplasms that can occur in any organ [11]. They most commonly occur in the gastrointestinal tract and lungs and rarely occur in other organs such as the thyroid, genitourinary system, and breast [12]. Their incidence in the breast is estimated to range between 1 and 5%, though it is difficult to obtain an accurate estimate since breast tumors are not routinely stained with neuroendocrine markers [11,13]. Breast neuroendocrine tumors (BNETs) belong to the greater family of breast neuroendocrine neoplasms (NENs). The World Health Organization (WHO) updated its classification of breast NENs in 2019 to include three main categories: well-differentiated BNETs, invasive breast cancers of no special type with neuroendocrine differentiation, and neuroendocrine carcinomas (NECs) [14].

Tumors associated with EAS are most commonly derived from the lung, with pulmonary NETs being the most common, followed by small-cell carcinoma and adenocarcinoma [6]. It has been estimated that BNETs account for less than 1% of tumors associated with EAS [6]. Unlike EAS in the lung, these are not usually associated with ACTH levels high enough to produce the classic CS symptoms [15]. However, patients with significant levels of ACTH tend to present acutely with hypokalemia, weight loss, abnormal glucose tolerance, and metabolic alkalosis [16]. Hypokalemia is the most common symptom and is seen in approximately 70% of cases [6,17]. This syndrome can arise at any stage of the disease and may be difficult to diagnose as it is often masked by symptoms of the primary tumor. The severity of these findings is dependent on the degree of ACTH secretion, also complicating diagnosis [15].

In this report, we describe a rare case of EAS derived from neuroendocrine carcinoma of the breast that was ultimately identified upon the development of severe hypokalemia. Written informed consent was obtained for the reporting of this case.

## 2. Case Report

A 39-year-old female presented with a rapidly enlarging, self-detected right breast mass. Physical examination bilaterally confirmed dense breast tissue with a large right breast mass in the upper outer quadrant, mobile from the chest wall with associated skin tethering. No axillary, cervical, and supra- or infra-clavicular adenopathy was palpated.

**Scheme 1:**
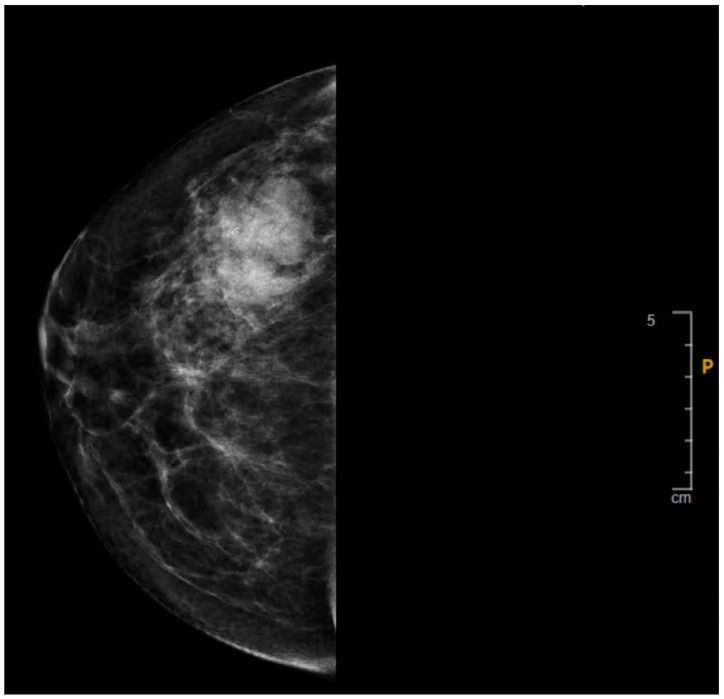
Right breast mammogram demonstrating an ill-defined mass of increased density containing several microcalcifications in the mid-outer right breast measuring 3.5 cm, BI-RADS 5.

**Scheme 2:**
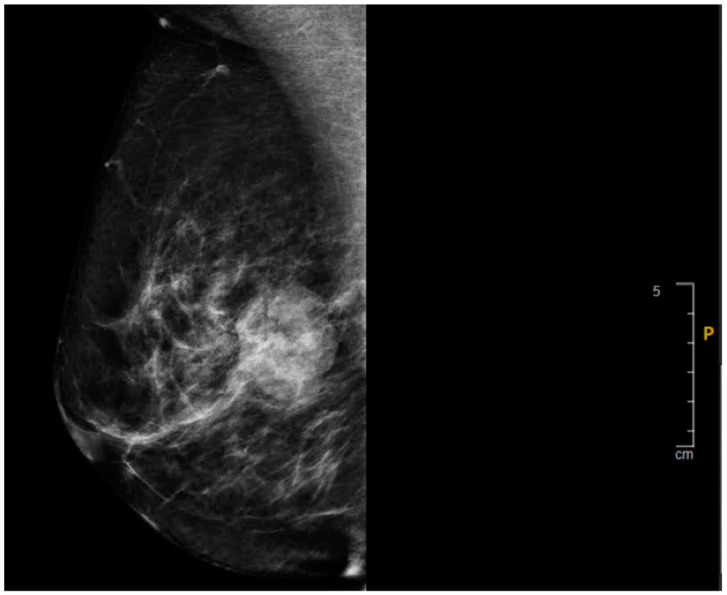
Right breast mammogram demonstrating an ill-defined mass of increased density containing several microcalcifications in the mid-outer right breast measuring 3.5 cm, BI-RADS 5.

**Scheme 3:**
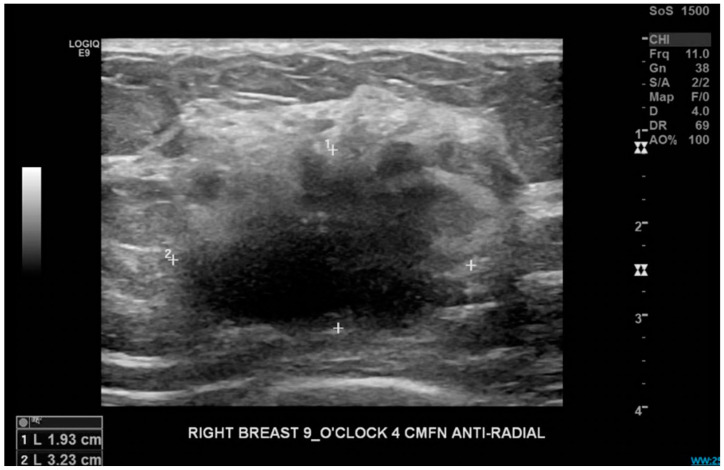
Right breast ultrasound demonstrating a 2 × 3 × 3 cm hypoechoic mass in the 9:00 position, BI-RADS 5.

Urgent imaging workup with bilateral mammogram demonstrated an ill-defined mass of increased density containing several microcalcifications in the mid-outer right breast measuring 3.5 cm (BI-RADS 5) (Scheme 1 and Scheme 2). A bilateral breast ultrasound confirmed a solid 2 × 3 × 3 cm hypoechoic mass in the 9:00 position of the right breast, 4 cm from the nipple (BI-RADS 5) with no pathologic lymph node abnormalities (Scheme 3). Initial core biopsy demonstrated “invasive poorly differentiated breast carcinoma with high-grade neuroendocrine features and necrosis.” Receptor status demonstrated estrogen receptor (ER) negativity, progesterone receptor (PR) positivity, and human epidermal growth factor receptor 2 (HER-2) negativity by immunohistochemistry. Further pathology review was requested given diagnostic uncertainty. Immunohistochemical assessment demonstrated positive staining for pancytokeratin, synaptophysin (SYN), chromogranin (CG), CD56, CK7 (partial), and TTF-1. Negative stains included cytokeratin 34BE, mammaglobin, GCDFP, GATA, CK20, and CK5/6. Repeat hormone receptor analysis confirmed ER negativity and showed weak focal staining of PR. Discussion at pathology consensus rounds raised the possibility of metastases from a lung primary given the diffuse, strong expression of TTF-1; however, according to the WHO classification of tumors 5th edition [14], neuroendocrine carcinoma of the breast should not be shown.

**Scheme 4:**
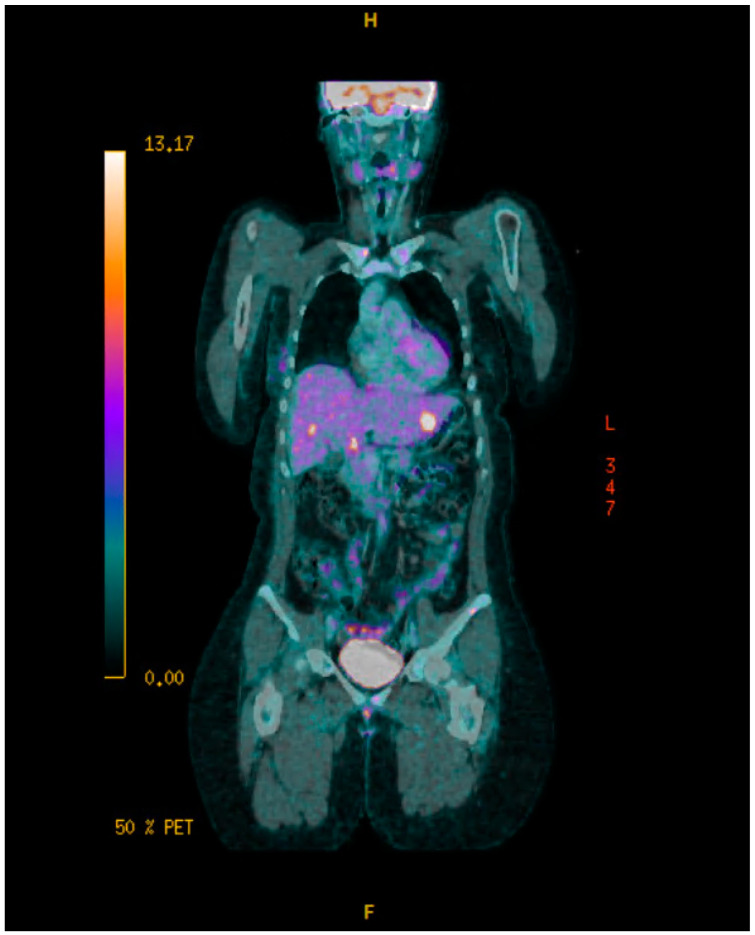
Initial staging PET-CT demonstrating hepatic metastases.

Staging imaging at diagnosis with computed tomography (CT) of the chest, abdomen, and pelvis and whole-body bone scan did not demonstrate any findings of metastatic disease; however, a subsequent positron emission tomography–computed tomography (PET-CT) scan two weeks later confirmed diffuse metastatic disease of the liver and bones (Scheme 4).

The patient’s past medical history was otherwise significant for hypothyroidism for which she was prescribed Synthroid. She had no surgical history. She was a non-smoker and used alcohol socially. Her family history was significant for a malignant brain tumor in her brother (of unknown type as per the patient) resulting in his death at age 11.

Given the metastatic nature of the disease, medical oncology was consulted and first-line chemotherapy (Cisplatin/Etoposide/Durvalumab) for treatment of invasive breast carcinoma with high-grade neuroendocrine features was initiated. After the completion of four cycles, restaging CT and PET-CT imaging demonstrated evidence of disease progression in both the primary site and metastatic lesions. Following this, the patient began to experience right-sided lower back pain overlying the iliac crest near a site of known bone metastasis.

Given the poor response to systemic therapy, the General Surgery service was re-consulted for consideration of a complete excision of the primary tumor for local control and a complete pathological analysis of the tumor. Given the metastatic nature of the disease and lack of axillary symptoms, surgical assessment of the lymph nodes was not indicated. The patient underwent an urgent right simple mastectomy that was uncomplicated. Final pathology demonstrated high-grade neuroendocrine carcinoma with large-cell cytological features present in a single 4.3 cm mass with extensive lymphovascular invasion. ER-negative (0%), PR focally positive (15%), and HER-2-negative (0%).

**Scheme 5:**
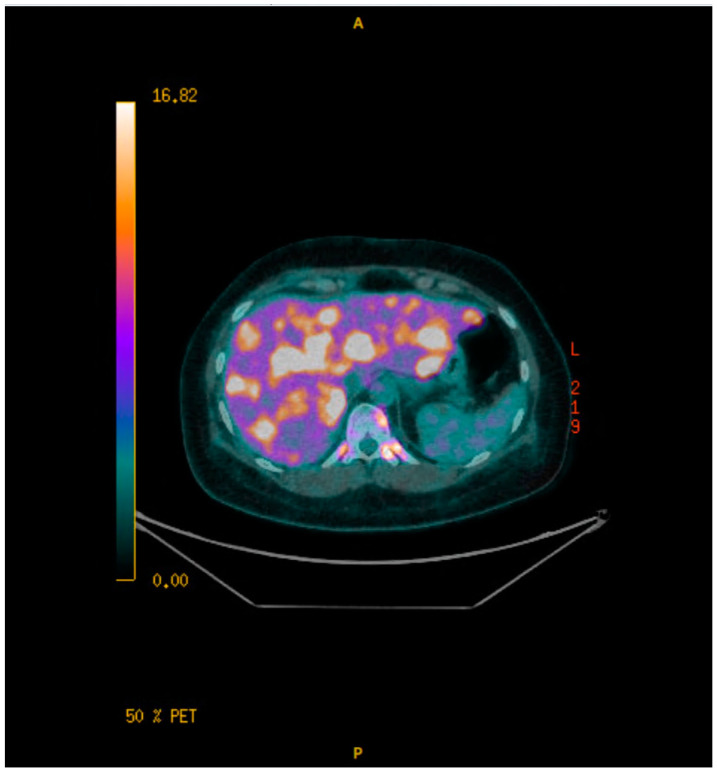
Repeat PET-CT demonstrating progression of metastatic disease.

Following mastectomy, a restaging PET-CT was completed, which showed the progression of metastatic disease (Scheme 5). Genetic testing was completed, confirming a PIK3CA mutation for which special access to Alpelisib (Piqray^®^), a PI3Kα inhibitor, was requested [18,19].

Once recovered from surgery, second-line therapy (Doxorubicin/Cyclophosphamide) was initiated, which the patient initially tolerated well but continued to experience severe pain from the bone metastases. After three cycles, the patient presented to the emergency department with swelling over her eyelids, trunk, and extremities, as well as pain at the base of her skull, dizziness, and headache. The potassium level was found to be 2.7 mmol/L, which had previously been confirmed to be normal. She received potassium supplementation in the emergency department and experienced a resolution of the dizziness. The patient was discharged home with potassium chloride supplementation. Repeat laboratory investigations a few days later confirmed persistent hypokalemia with a potassium of 2.5 mmol/L for which the patient was admitted to the medical oncology service.

**Scheme 6:**
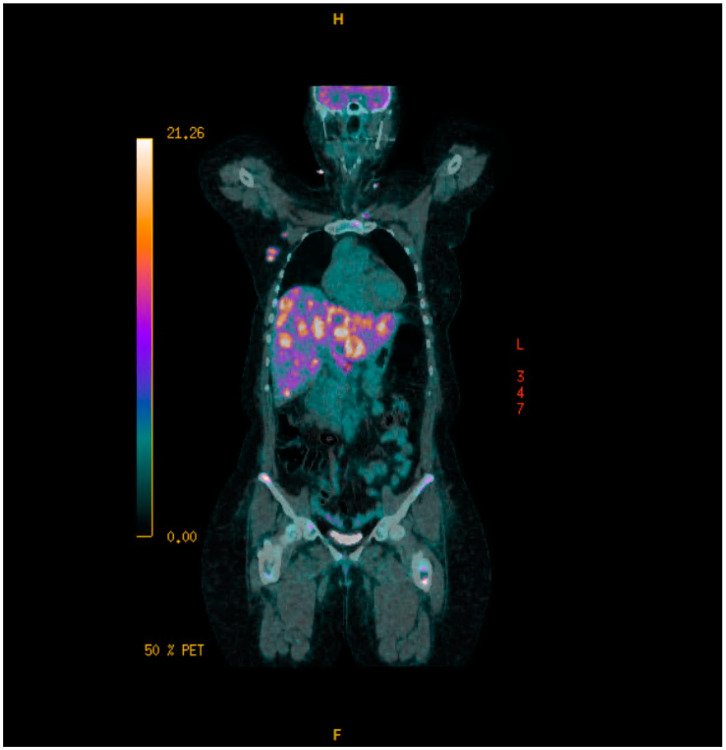
Repeat PET-CT demonstrating progression of known hepatic and skeletal metastases with new lymph node involvement.

**Scheme 7:**
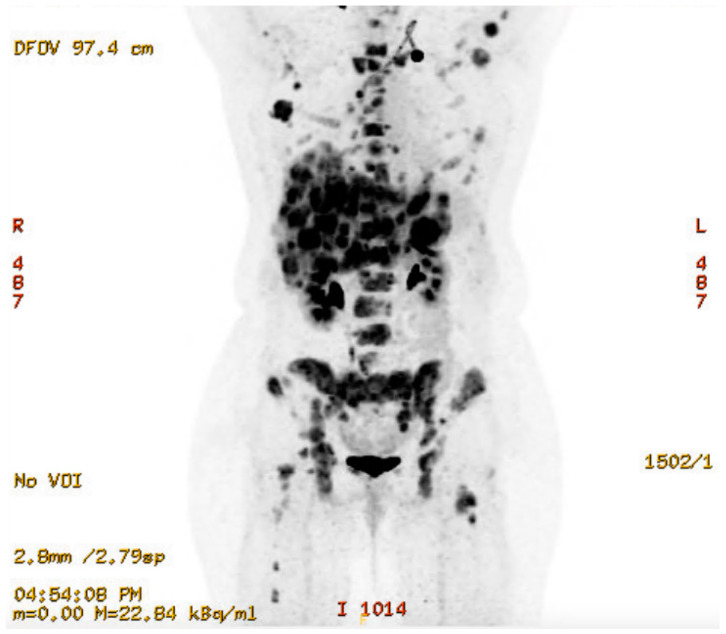
Repeat PET-CT demonstrating progression of known hepatic and skeletal metastases with new lymph node involvement.

Repeat PET-CT was completed soon after admission, which again showed progression of the liver and bone metastases and new metastases to lymph nodes (Scheme 6 and Scheme 7). Access to Alpelisib therapy was denied by her insurance company and therefore third-line therapy (Nab-Paclitaxel) was initiated.

The patient continued to experience refractory hypokalemia despite repeated oral and intravenous potassium supplementation. A dedication review confirmed no potassium-lowering interactions, and gastrointestinal losses were not experienced. Ongoing workup demonstrated a morning cortisol level of 2454 nmol/L in the absence of exogenous steroid administration. At this time, a diagnosis of EAS was suspected. On repeat assessment, morning cortisol levels were 2693 nmol/L and therefore testing directed at diagnosing CS was performed. A 24 h urinary free cortisol test found extremely elevated cortisol levels at 27,387 nmol/L (28–276.5 nmol/L). Repeat confirmatory 24 h urine-free cortisol testing showed progressive ongoing extreme elevation at 72,402.7 nmol/L (28–276.5 nmol/L) and the diagnosis of CS was established. Plasma ACTH was 76.8 pmol/L (1.6–13.9 pmol/L), consistent with excess ACTH due to her BNET confirming EAS. Clinically, she did not demonstrate strong features of CS with no further episodes of dizziness, euglycemia, and absence of hypertension. Given the burden of the disease, resection of the BNET as a means of controlling ectopic ACTH production was not possible and therefore medical therapy with Ketoconazole was initiated for the management of CS.

Despite this, she required ongoing potassium replacement and spironolactone therapy for the management of refractory hypokalemia (Potassium = 2.8 mmol/L, 3.5–5.0 mmol/L). Ketoconazole was titrated to a near-maximal dose; however, despite this, the patient continued to experience progressive, severe symptoms of CS. Concern for Ketoconazole-associated hepatotoxicity, in the setting of high-burden metastatic liver disease and poor response to initial doses, raised the question of further steps to urgently control hypercortisolism. Given the severity of the disease and significant tumor burden, urgent bilateral adrenalectomy was recommended as a life-preserving emergency treatment. This was completed laparoscopically without complication. Postoperative normalization of hypokalemia and hyperglycemia was achieved.

Following surgery, she received steroid replacement therapy for treatment of iatrogenic adrenal insufficiency and VTE prophylaxis given the prothrombotic effect of CS. Steroids were tapered postoperatively with the goal of achieving physiologic maintenance dosing. Prednisone (25 mg daily) with a planned taper to physiologic dosing in combination with Fludrocortisone (0.1 mg daily) was given. The patient was given instruction on symptoms of a potential adrenal crisis, stress dosing of steroid therapy, and a rescue preparation of intramuscular Solucortef (100 mg) to be used as needed. The patient was successfully discharged home, relieved to be reunited with her young family, while awaiting additional systemic therapy for her primary carcinoma. She reported minimal postoperative pain and significant improvement in her quality of life. During this time, she was followed closely by Endocrinology with electrolyte monitoring.

One month later, the patient was readmitted with hyponatremia and adrenal insufficiency likely precipitated by further progression of the disease. During this time, she experienced a fall after which examination demonstrated no acute injuries. She was treated with stress dosing of IV hydrocortisone and Sodium Chloride. The following evening, she developed acute onset shortness of breath and tachycardia with progressive rapid deterioration resulting in cardiac arrest. Despite cardiopulmonary resuscitation, the return of spontaneous circulation was not achieved. An autopsy was not completed to determine the cause of death. Cardiac complications associated with prolonged CS versus pulmonary embolism were considered. Alternate coverage for Alpelisib therapy was obtained during this time frame; however, the patient passed away before she was able to receive this treatment.

## 3. Discussion

**Figure 1:**
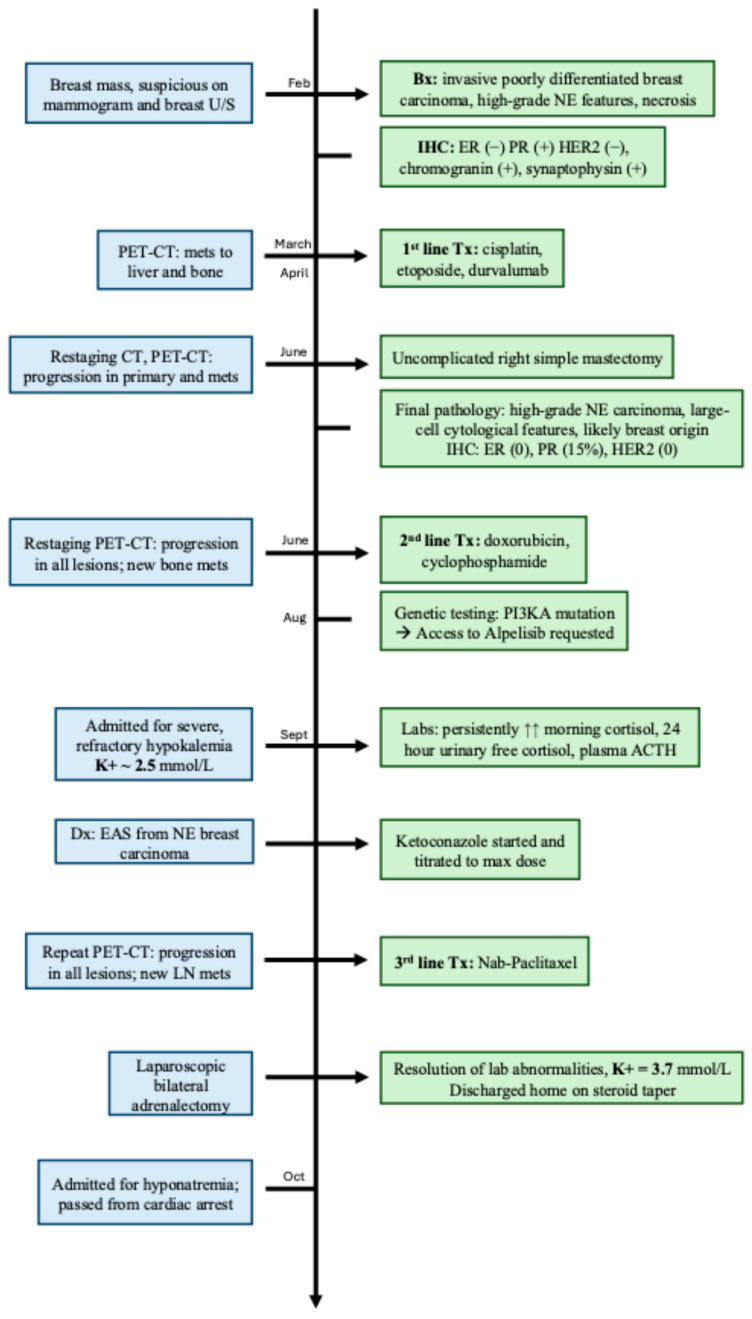
Timeline of investigations and therapies. U/S: Ultrasound, Bx: Biopsy, NE: Neuroendocrine, IHC: Immunohistochemistry, ER: Estrogen receptor, PR: Progesterone receptor, HER2: Human epidermal growth factor receptor 2, PET-CT: Positron emission tomography–computed tomography, Tx: Treatment, K: Potassium, ACTH: Adrenocorticotropic hormone, Dx: Diagnosis, EAS: Ectopic adrenocorticotropic hormone syndrome, LN: Lymph node.

In this case report, we describe the management of a rare diagnosis of metastatic neuroendocrine breast carcinoma resulting in the paraneoplastic syndrome known as EAS (Figure 1).

Primary BNETs can be difficult to diagnose because they have a similar clinical presentation to other types of breast cancer, including localized nodularity, axillary adenopathy, and nipple symptoms [20]. While these tumors are rarely found in the breast, it is even more rare for them to develop a paraneoplastic syndrome of hormone hypersecretion, such as in EAS, in this location [21]. Ectopic release of other compounds from BNETs, including norepinephrine and calcitonin, has been reported [22,23]. Unlike the case described here, the level of secretion of ACTH from BNETs in EAS is not typically significant enough to produce symptoms of CS [24].

The key clinical feature of this case leading to an accurate diagnosis of EAS was refractory hypokalemia. This finding is commonly associated with EAS from cancers of multiple organs, including the breast [17]. One study found that 70% of patients in a population with histologically proven EAS exhibited severe hypokalemia, with a median value of 2.9 mmol/L [17]. Multiple case reports of EAS from BNETs report hypokalemia as a common abnormality among patients [15]. Thus, this syndrome should always be considered when managing a patient with breast cancer and concomitant refractory hypokalemia.

Currently, there are no defined guidelines for diagnosing BNETs. Neuroendocrine biomarkers including CG and SYN are most commonly used for histological identification but are somewhat nonspecific and not routinely tested for tumors in this location [21]. In a study assessing 224 cases of neuroendocrine-differentiated breast cancer, Lai et al. demonstrated that a lower level of expression of the neuroendocrine markers CG, SYN, or CD56 was correlated with decreased overall survival and higher rates of recurrence [25].

Reports of typical hormone receptor status for these tumors have been variable. Smaller studies and case reports have shown that BNETs tend to be estrogen and progesterone receptor-positive and HER2-negative [26,27,28]. However, a recent retrospective cohort analysis of 1389 BNETs from the American National Cancer Database found that BNETs were more often hormone receptor-negative compared to a matched cohort of invasive ductal carcinoma (IDC) [21]. They postulate that given their large cohort size, it is likely that the majority of BNETs are in fact hormone receptor-negative.

The prognosis for BNETs is inconclusive due to limited reports in the literature and variable study inclusion criteria [29]. One retrospective study assessing 142 patients with neuroendocrine breast tumors found that neuroendocrine differentiation itself was a poor prognostic factor, independent of other factors such as patient age and tumor staging [27]. Wei et al. also found that BNETs were more aggressive than IDC and were more likely to display local and distant recurrence [26]. This was echoed by Martinez et al., who found that BNETs tend to present at more advanced stages and behave more aggressively than IDC, resulting in decreased overall survival [21]. Conversely, it has been hypothesized that the presence of neuroendocrine differentiation allows for more targeted treatment and thus better outcomes [25].

In the absence of clear guidelines for the management of BNETs, most reports include the use of a multimodal approach to treatment involving surgery, radiation, and chemotherapy. Chemotherapy regimens largely involve platinum agents and Etoposide and are thus similar to those used for small-cell lung cancer [16]. In general, higher-grade NETs tend to be more responsive to chemotherapy than low-grade NETs due to their rapid cell division rate making them more susceptible to cytotoxic agents [30]. Given the typically negative hormone receptor status of these BNETs and the fact they tend to present at later stages, a higher proportion of these cases tend to be treated with systemic therapy in comparison to IDC matched cohorts [21]. For HER-2 negative cases that do demonstrate hormone receptor positivity, Wei et al. demonstrated that patients had better outcomes when treated with endocrine therapy in combination with radiation as compared to chemotherapy, though this difference was not statistically significant [26].

Due to a scarcity of reports in the literature, no specific management strategies have been developed for treating EAS resulting from BNETs [16,24]. Although the definition of intense or severe hypercortisolism remains imprecise, associated complications are extremely common with significant impact on overall prognosis and mortality. For this reason, severe EAS should be considered an “endocrine emergency”. North American Endocrine Society guidelines recommend the resection of primary lesions (pituitary, ectopic, or adrenal), when possible, as first-line management for CS [31]. In the case of ectopic ACTH secretion secondary to an underlying malignancy, important factors to consider include the intensity of hypercortisolism, the patient’s general condition, comorbidities, and the resectability status of the ACTH-secreting tumor. The ideal treatment is complete excision of the secreting tumor [32]. However, when this is not possible, a multidisciplinary team should implement an individualized therapeutic strategy including monitoring for complications of CS such as venous thromboembolism, infection, electrolyte disturbances, and tumor progression.

Medical therapies for hypercortisolism can be categorized into three groups based on their mechanism of action, including steroidogenesis inhibitors, neuromodulators of ACTH release, and glucocorticoid receptor-blocking agents. Steroidogenesis inhibitors reduce cortisol levels through adrenolytic activity and/or direct enzymatic inhibition. Neuromodulatory compounds modulate ACTH release from a pituitary tumor and glucocorticoid antagonists block cortisol action at its receptor. In the management of EAS, steroidogenesis inhibitors are the agent of choice. These include drugs such as Mitotane, Metyrapone, Aminoglutethimide, and Ketoconazole. Ketoconazole is the best tolerated agent and is an effective monotherapy in about 70% of patients [33]. In cases of extreme hypercortisolism, high or maximal dose Ketoconazole therapy is required. Endocrinologists must be wary of its associated hepatotoxicity, which can be compounded by pre-existing liver disease or dysfunction associated with hepatic metastases; thus, its utility in these cases may be limited [32].

When medical management is not sufficient, bilateral adrenalectomy should be considered given its highly effective and immediate response [31]. It is important to recognize that bilateral adrenalectomy in this population carries a significant risk of surgical mortality and intraoperative complications, which are more frequent with EAS than other forms of CS [34]. Complications of adrenalectomy in EAS include myocardial infarction, internal bleeding, injury to other organs, poor wound healing, surgical site infections, and postoperative thromboembolic events [32]. When possible, the laparoscopic approach is preferred to reduce the morbidity of the procedure. Additional consideration must be given to the treatment of iatrogenic adrenal insufficiency following surgery, with education on the recognition of outpatient adrenal crisis and stress dosing of steroid therapy.

Through a retrospective review of this case, we note that, for the patient’s benefit, the diagnosis of BNET was made at the time of initial pathologic assessment, allowing for the initiation of appropriate systemic therapy early in her treatment pathway. Given the rapid progression of the disease, the treatment team astutely sought further pathological analysis and mutational assessment to confirm the diagnosis and allow for more targeted therapeutic options. It remains unclear whether the use of targeted therapy (Alpelisib) would have resulted in improved outcomes because, due to difficulties obtaining funding for this drug, the patient succumbed to her disease before it was available to her. Secondly, the team was quick to consider and identify the cause of the patient’s refractory hypokalemia, resulting in an efficient diagnosis of EAS followed by subsequent management.

Overall, this case illustrates the significant challenges associated with diagnosing and managing EAS resulting from BNETs. While bilateral adrenalectomy is appropriately not considered first-line therapy, in refractory cases, it remains an important therapeutic option. In this patient’s case, while not significantly impacting overall survival, adrenalectomy was completed achieving the goal of reducing morbidity associated with EAS and improving the quality of life for this patient while awaiting additional therapy.

## 4. Conclusions

EAS resulting in CS secondary to breast neuroendocrine carcinoma is a rare diagnosis that poses many challenges relating to its diagnosis and management. Further research is needed to inform the development of treatment guidelines to improve outcomes in the future. While patient survival is dependent upon the underlying disease process, laparoscopic bilateral adrenalectomy is an accepted, definitive treatment option for BNET-associated EAS [35].

## Data Availability

The data presented in this study are available on request from the corresponding author due to patient anonymity.

## Abbreviations

The following abbreviations are used in this manuscript in the order they appear:

CS	Cushing’s Syndrome
ACTH	Adrenocorticotropic hormone
EAS	Ectopic adrenocorticotrophic hormone syndrome
POMC	Pro-opiomelanocortin
NETs	Neuroendocrine tumors
BNETs	Breast neuroendocrine tumors
NENs	Neuroendocrine neoplasms
WHO	World Health Organization
NECs	Neuroendocrine carcinomas
BI-RADS	Breast Imaging-Reporting and Data System
ER	Estrogen receptor
PR	Progesterone receptor
HER-2	Human epidermal growth factor receptor 2
CG	Chromogranin
PET-CT	Positron emission tomography–computed tomography
PSOGI	Peritoneal Surface Oncology Group
IDC	Invasive ductal carcinoma
